# Renal denervation improves cardiac function by attenuating myocardiocyte apoptosis in dogs after myocardial infarction

**DOI:** 10.1186/s12872-018-0828-y

**Published:** 2018-05-08

**Authors:** Li Wang, Lijun Song, Chao Li, Qiaoli Feng, Mengping Xu, Zhuqing Li, Chengzhi Lu

**Affiliations:** 1 First Center Clinic College of Tianjin Medical University, Tianjin First Center Hospital, 24 Fukang Road, Nankai District, Tianjin, 300192 China; 2 Department of Cardiology, Tianjin First Center Hospital, Tianjin, China; 3 Department of Digestion, Tianjin First Center Hospital, Tianjin, China

**Keywords:** Renal denervation, Heart failure, Myocardial infarction, Myocardial apoptosis

## Abstract

**Background:**

Myocardial apoptosis is important in the pathogenesis and progression of myocardial infarction-induced heart failure (MI-HF). Renal sympathetic denervation (RDN) has become a promising therapeutic strategy for the treatment of HF. Previous studies have shown that RDN could improve heart function Yao et al. (Exp Ther Med 14:4104-4110, 2017). However, whether and how RDN regulates myocardial apoptosis in MI-HF is unclear. This study sought to evaluate the effects of RDN on cardiac function and apoptosis-related gene expression in MI-HF dogs.

**Methods:**

Eighteen healthy mongrel dogs were randomly divided into control group(*n* = 6), model group(*n* = 6) and treatment group(*n* = 6). MI-HF was established in model group and treatment group by anhydrous alcohol embolization, after heart failure dogs in the treatment group and model group proceeded bilateral renal artery ablation and bilateral renal arteriography, respectively. The cardiac function parameters were evaluated by echocardiographic; the serum NT-BNP level was detected by ELISA; the degree of myocardial fibrosis was observed through masson staining; the expression of MMP-2, MMP-9 in the cardiac were got by immunohistochemistry. TUNEL method was used to observe cardiomyocyte apoptotsis and calculate the apoptosis index (AI). Relative expression of Bcl-2 and Bax, Caspase3 and GRP78 were detected using RT-PCR and Western Blot. Renal artery H&E staining and serum creatinine were conducted to access the efficacy and safety of RDN.

**Results:**

Four weeks after RDN, the LVEDD, LVESD and LVEDP decreased, and the LVEF and LVSP increased in the treatment group compared with those in the control group (all *P* < 0.05). Moreover, NT-BNP, an indicator of cardiac function was decreased. Additionally, MMP-2 and MMP-9 levels in the myocardium decreased significantly in the treatment group. Furthermore, the levels of Bax, and caspase 3 decreased, while the level of Bcl-2 increased. Thus, myocardial apoptosis was attenuated in RDN treated dogs. We also found that the level of GRP78 which is activated in response to endoplasmic reticulum (ER) stress, was decreased. However, serum creatinine levels were not significantly different between the RND-treated dogs and the control dogs.

**Conclusion:**

Cardiac function was improved by RDN treatment through regulating apoptosis and ER stress in cardiomyocytes in dogs after MI.

## Background

Myocardial infarction is a leading cause of death in the world [1]. Heart failure (HF) is a common complication of acute myocardial infarction (AMI) and is characterized by cardiac dysfunction along with autonomic imbalance. HF is a serious condition with poor survival rates. Myocardial apoptosis is important in the pathogenesis and progression of MI-induced HF. The local adverse stimulus after MI induces myocyte apoptosis [2] and promotes the deterioration of cardiac function and ventricular remodeling. Loss of myocardial cells is associated with impaired heart function.

A previous study suggested that sympathoexcitation plays a critical role in the pathogenesis and progression of HF after MI. Thus, inhibiting the adverse stimulus of sympathetic overactivity after MI may attenuate myocyte apoptosis and improve cardiac function, which may have clinical significance in the treatment of HF. Catheter-based renal sympathetic denervation (RDN) selectively reduces both renal sympathetic efferent and afferent nerve activity, which has become a hot topic in the field of HF treatment [3, 4]. Whether and how RDN regulates apoptosis in MI-HF is unclear. Thus, the aim of the study was to evaluate the effects of RDN on cardiac function and apoptosis-related gene expression in MI-HF dogs.

## Methods

### Animal model

Mongrel dogs (male = 7, female = 11) weighing between 15 and 18 kg were purchased through the Experimental of Animal Care Center of Tianjin Medical University, there was no gender difference observed in our study. All the experiments were conducted in accordance with the guide for the Care and the Use of Laboratory Animials and were approved by the ethical committee of Tianjin Medical University.

### Experimental groups

All experimental dogs were randomly assigned into three groups: (1) control group (*n* = 6) received only a coronary angiogram; (2) model group (*n* = 6) that underwent an established procedure to induce MI and four weeks later underwent a renal arteriogram; (3) treatment group (*n* = 6) first underwent MI-inducing procedure and then underwent RDN four weeks later.

### Establishment of MI model

Dogs in model and treatment groups were anesthetized with sodium pentobarbital (30 mg/kg IV), intubated, and ventilated using a respirator with room air supplemented with oxygen. Continuous ECG monitoring was carried out. After femoral artery access was established and 1000 IU of heparin was injected, 0.2 ml of anhydrous alcohol was injected distally to the first diagonal of the left anterior descending coronary (LAD) as described in a previous study, resulting in left ventricular (LV) damage.

### Renal sympathetic denervation

In the treatment group dogs, the ablation electrode (6F saline irrigated catheter tip) was inserted into each artery via the fermoral artery under fluroscopy, and radiofrequency (RF) energy was applied to the endothelial lining with an irrigation flow rate of 12 ml/min. The temperature and power of the radiofrequency ablation instrument was set at 43 °C and 10 W.The target sites were in different directions,lasted for at least 90 s.The catheter was then retreated1–2 cm to generate another ablation location. This procedure was repeated four times in each renal artery, and then, the similar RF energy was applied to the contralateral renal artery.

### Transthoracic echocardiography

All of the dogs underwent transthoracic ecchocardiography at baseline, 4 weeks after MI and 4 weeks after RDN (CX50, Philips, Netherlands). The left ventricular ejection fraction (LVEF), left ventricular end-systolic dimension (LVESD), left ventricular end-diastolic dimension (LVEDD), left ventricular systolic pressure (LVSP) and left ventricular end-diastolic pressure(LVEDP) were recorded. Three consecutive cardiac cycles were observed, and the average values were recorded as the final cardiac parameters. The LVEF was calculated by the formula: (LVVmax- LVVmin) / LVVmax.

### Evaluation of plasma NT-BNP and creatinine level

Venous blood were collected in vacutainers for the NT-BNP and Cr assays. Samples for the NT-BNP and Cr assays were centrifuged at 3000 g for 10 min at 4 °C, and the plasma was separated kept in microtubes and stored at − 70 °C until assay. NT-BNP and Cr levels were examined by using enzyme-linked immunosorbent assay (ELISA). All assays were performed twice.

### Histological evaluation

After perfusion with ice-cold PBS, the hearts were cut and fixed in 4% phosphate buffered formalin for 48–72 h at 4 °C, subsequently, the tissues were then dehydrated and embedded in paraffin. Infarcted myocardial tissue was flash-frozen by liquid nitrogen then stored at − 80 °C. Masson’s trichrome staining were performed to detect cardiac fibrosis.Connective tissue was differentiated on the basis of its color. Immunohistochemical staining was performed using the Power VisionTM two-step method. The sections were stained with MMP-2(polyclonal rabbit anti-MMP-2 antibody,Abcam, USA) and MMP-9(polyclonal rabbit anti-MMP-9 antibody Santa Cruz, USA). Immunohistochemical score (IHS) was utilized. This method has been shown to approximate data generated from image analysis-based scoring systems as described in a previous study [5]. HE staining was used to detect the renal artery.

### TUNEL staining

TUNEL staining was performed using a commercial kit (In Situ Cell Death Detection FITC Kit or TMR red, Roche). Myocardial tissues were fixed in 4% paraformaldehyde and dehydrated with ethyl alcohol. Paraffin sections were incubated with anti-α-actin in antibodies (1:200 dilution; Sigma-Aldrich) in a humidified chamber. The cells that exhibited condensed nuclei with an irregular form or nuclei split into green particles were considered to be TUNEL-positive cells. The apoptotic index (AI) was calculated according to the following formula: AI = (number of apoptotic cells/total number of nuclei) × 100%.

### Western blot

Protein expression levels of Bcl-2 (MDL, China), Bax (MDL, China), caspase 3 (MDL, China), and GRP78 (MDL, China) were assessed by western blotting. All antibodies were applied according to the manufacturer’s instructions. The integrated optical densities of the protein bands were obtained by an imaging system.

### RT-PCR

mRNA levels were assessed by using quantitative real-time reverse transcription polymerase chain reaction (RT-PCR). Total RNA was extracted from myocardial tissue. Purified RNA was quantified, and cDNA was synthesized using an I-script cDNA synthesis kit (Bio-Rad).

TaqMan primers from Life Technologies were used in the quantitative real-time RT-PCR. The sequences for the primers are summarized in supplemental Table 1. The values were corrected based on ß-actin levels, and the final mRNA level was calculated using the formula *x*=2^-ΔΔCT^, where *x* is the fold change relative to the control.

### Statistical analysis

The data were presented as the mean ± SD. Group comparisons were subjected to analysis of variance (ANOVA), followed by the least significant difference (LSD) test to identify differences among various groups,and a probability value < 0.05 was required for statistical significance(version 20.0 SPSS).

## Results

One dog in the treatment group died 1 day after AMI because of ventricular fibrillation, and one dog in the model group died of HF 30 days after AMI; there were no deaths in the normal group.

### Baseline parameters

Dogs in each group underwent assessment of the LVEDD, LVESD, LVEF, LVEDP, LVSP and HR before MI (shown in in supplemental Table 2), and no significant baseline differences were found among the three groups (all *P* > 0.05).

### RDN improved the function of failing hearts

Four weeks after MI, and compared to baseline data, the LVEDD, LVESD and LVEDP were significantly increased (all *P* < 0.05) while the LVEF and LVSP values were both reduced in the model and treatment groups (both *P* < 0.05). Importantly, 4 weeks after RDN, parameters such as LVEDD, LVESD, LVEF, LVEDP and LVSP were significantly improved in the treatment group compared with those in the model group (all *P* < 0.05), but the LVEDD, LVESD, and LVEDP in the treatment group were still higher than the baseline parameters (all *P* < 0.05), and LVEF lower than baseline group (all P < 0.05) (shown in Table 1).

**Table 1:** Data on cardiac function before and after RDN

	Model group (*n* = 5)		Treatment group (*n* = 5)	
	Pre-sham	Post-sham	Pre-RDN	Post-RDN
LVEDD (mm)	38.17 ± 1.92^*^	39.54 ± 1.89^*^	37.70 ± 3.04^*^	35.36 ± 2.63^*#^
LVESD (mm)	29.80 ± 1.42^*^	32.64 ± 4.51^*^	28.60 ± 3.21^*^	26.08 ± 3.89^*#^
LVEF (%)	41.13 ± 2.88^*^	36.78 ± 3.44^*^	39.86 ± 3.47^*^	43.80 ± 2.66^*#^
LVEDP (mmHg)	17.67 ± 6.50^*^	22.40 ± 5.90^*^	20.20 ± 6.54^*^	13.20 ± 3.19^*#^
LVSP (mmHg)	103.80 ± 12.70^*^	93.18 ± 6.96^*^	102.40 ± 14.36^*^	109.8 ± 14.53^#^

Note: pre-RDN denotes 4 weeks after MI; values are presented as the mean ± SD. ^*^P < 0.05 vs. baseline data; ^#^*P* < 0.05 denotes the comparison between the model group and the treatment group

**Fig. 1:**
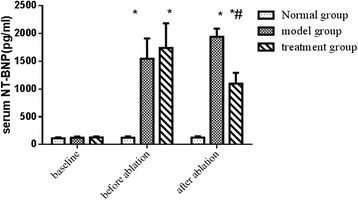
Changes in NT-BNP. Note: ^*^ *P* < 0.05 vs. baseline data; ^#^ *P* < 0.05 indicates the post-RDN comparison between the model group and the treatment group

At baseline, there were no differences in NT-BNP levels among the normal group, model group and treatment group. After myocardial infarction, NT-BNP levels in the model and treatment groups were significantly higher than those in the normal group (*P* < 0.05). However, after ablation, the NT-BNP level was significantly decreased in the treatment group compared with that in the model group (*P* < 0.05) (shown in Fig. 1).

### RDN suppressed cardiac fibrosis

**Fig. 2:**
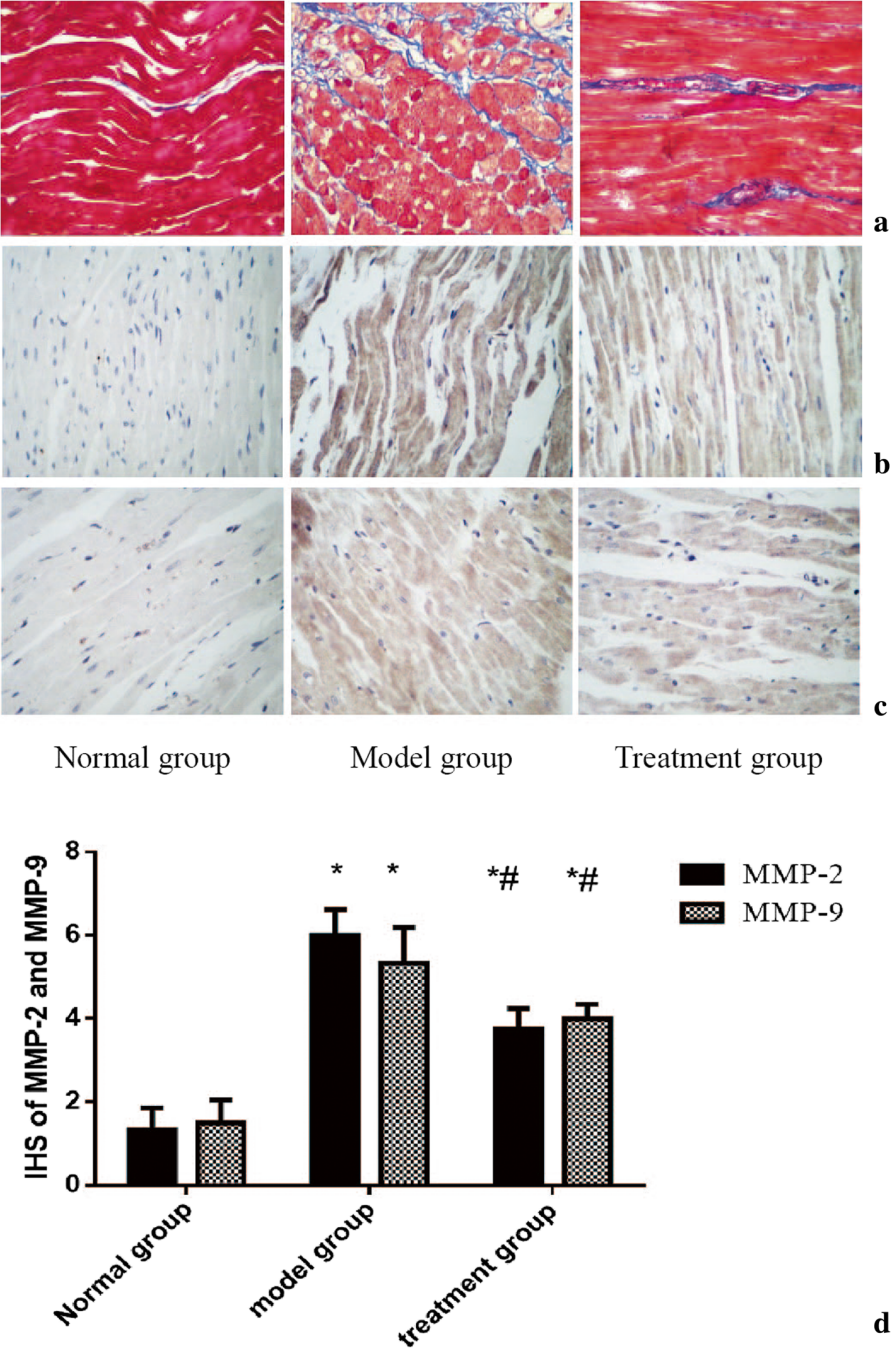
**a** Heart sections underwent Masson’s trichrome staining (magnification 400 X) to distinguish myocardial tissue (red) from fibrotic tissue (blue). In the normal group, the size of the myocardial cells was normal, and the muscle fibers were regular and no significant pathological changes were observed. In the model group, the cardiomyocytes were looser and the collagen fibers were significantly thickened and exhibited a net-like shape, and were infiltrated with a small amount of inflammatory cells. In the treatment group, the arrangement of myocardial cells was ordered, but there were still fibrotic changes compared with the normal group. **b** MMP-2 immunohistochemical staining of the infarct zone (400×). **c** MMP-9 immunohistochemical staining of the infarct zone (400×). **d** IHS of MMP-2 and MMP-9. Note: * *P* < 0.05 vs. the normal group; ^#^ *P* < 0.05 indicates the comparison of pre- and post-RDN between the model group and the treatment group

Following Masson’s trichrome staining, the sections presented different colors in different regions, with the blue color indicating fibrosis. As shown in Fig. 2A, staining for collagen showed an increase in intercellular space in both the model and treatment groups. After RDN, the level of myocardial fibrosis in the treatment group was significantly improved compared with the model group, and the arrangement of the myocardial cells was neat, however there was still fibrotic change compared with the normal group. Similar to the result observed with Masson’s staining, the MMP-2 (Fig. 2B) and MMP-9 (Fig. 2C) expression levels in the treatment group were significantly decreased compared with those in the model group but were still higher than those in the normal group, as shown in Fig. 2D.

### RDN suppressed cardiomyocyte apoptosis and reduced ER stress in cardiomyocytes

**Fig. 3:**
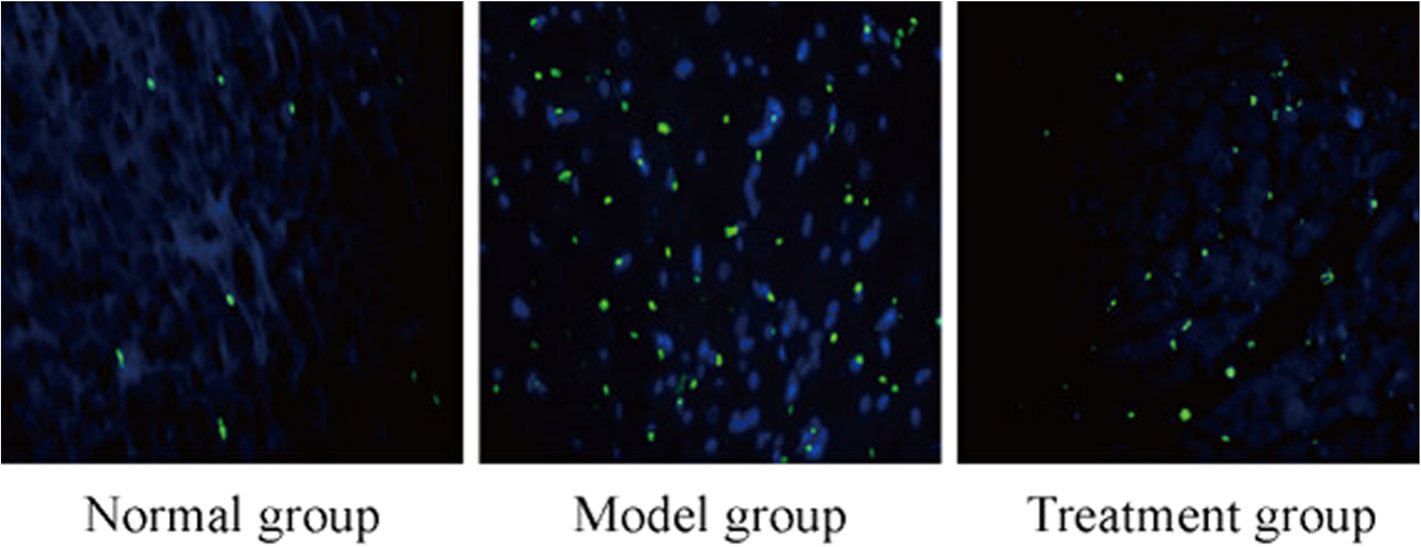
The AI in the normal, model and treatment groups was 2.30 ± 1.59, 30.26 ± 6.94 and 20.20 ± 4.00, respectively. Compared with the normal group, the optical density of TUNEL-positive cells was markedly increased in the model group and the treatment group. Compared with the model group, RDN treatment significantly attenuated cardiomyocyte apoptosis

The apoptosis index (AI), calculated as apoptotic cell/normal cell× 100, was used to estimate the degree of cardiomyocyte apoptosis, The AI in the normal, model and treatment groups was 2.30 ± 1.59, 30.26 ± 6.94 and 20.20 ± 4.00, respectively. Representative TUNEL staining is shown in Fig. 3. Compared with normal group, the optical density of TUNEL-positive cells was markedly increased in model and treatment groups (*P* < 0.01). and compared with the model group, RDN treatment significantly attenuated cardiomyocyte apoptosis (*P* < 0.01).

**Fig. 4:**
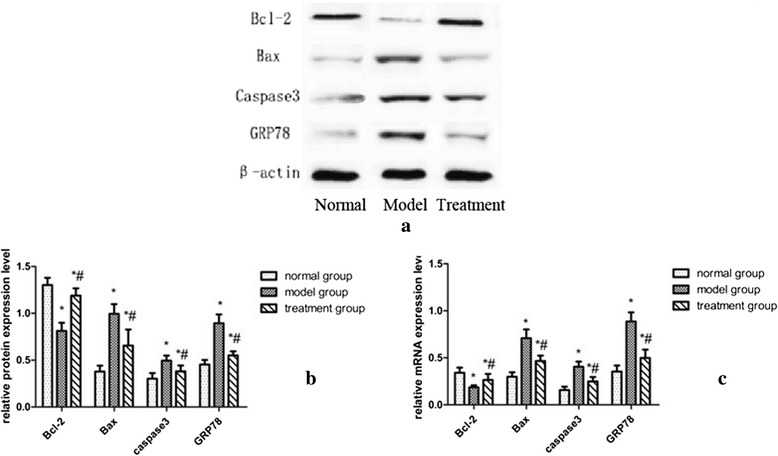
Effect of RDN on cardiomyocyte apoptosis and ER stress. **a**. Western blotting analysis of Bcl-2, Bax, caspase 3, and GRP78. **b** Quantitative analysis of the western blots. **c** mRNA expression levels of Bcl-2, Bax, caspase 3 and GRP78. Note: * *P* < 0.05 vs. the normal group; ^#^ *P* < 0.05 indicates the post-RDN comparison between the model group and the treatment group

The western blotting and RT-PCR analysis of Bcl-2, Bax, caspase 3 and GRP78 is presented in Fig. 4. Quantitative analysis showed that the model group had significantly increased expression levels of caspase 3, Bax, and GRP78 compared with the normal group (*P* < 0.01), whereas the level of Bcl-2 decreased markedly(*P* < 0.05). RDN significantly suppressed the upregulation of caspase 3, Bax and GRP78 and the downregulation of Bcl-2 protein and mRNA expression compared with the model group (*P* < 0.01).

### The safety of RDN

**Fig. 5:**
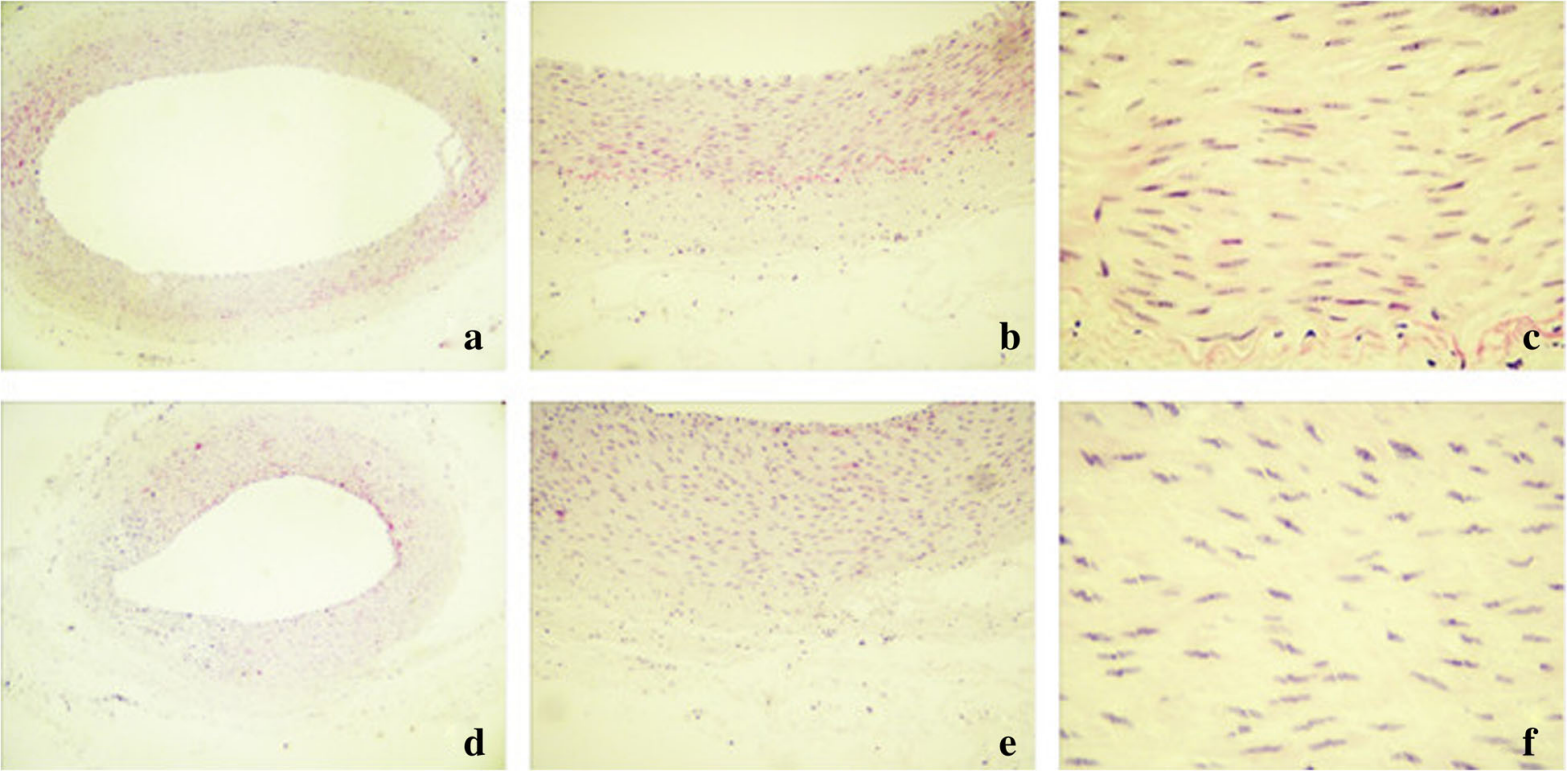
HE staining of the renal artery. Normal group (**a**-**c**): no damage to the vascular wall, intima or media, or to adventitial nerve fiber integrity (**a**: 40 X; **b**: 100 X; **c**: 400 X). Treatment group (**d**-**f**): no damage to the intima of the vessel wall, but the adventitial nerve fibers were destroyed (**d**: 40 X; **e**: 100 X; **f**: 400 X)

After RDN in the treatment group, no damage in the intima of the vessel wall was detected,

but the adventitial nerve fibers were destroyed, as shown in Fig. 5.

### No change in Cr levels before and after RDN

**Fig. 6:**
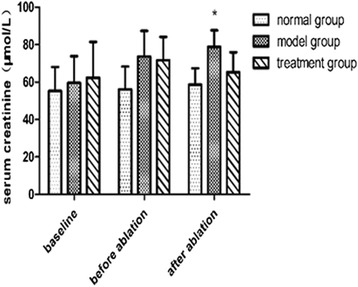
Change in Cr levels in different groups. Note: * *P* < 0.05 vs. the normal group

At baseline, there were no significant differences in Cr levels among the three groups. After building MI induced heart failure, there were still no markedly differences among three groups. Besides, there was no significant change after RDN in the treatment group, as shown in Fig. 6.

## Discussion

Increased sympathetic nerve activity is a primary characteristic of patients with chronic heart failure (CHF). Increases in sympathetic nerve activity enhance the release of catecholamines, such as norepinephrine, in the heart. Stimulation of β-adrenergic receptors induces cardiac myocyte apoptosis and fibrosis in the heart [6].Prolonged stimulation of the β-adrenergic neurohormonal axis has been shown to contribute to the progression of CHF and mortality in both animal models and humans. Amount of studies, whether clinical studies or animal studies showed that RDN could improve cardiac function in heart failure patients or animals by decreasing sympathetic nerve activity [7–11]. Our research group also showed that RDN improved cardiac function by decreasing cardiac oxidative stress [12]. Besides, previous study demonstrated that RDN leads to a significant improvement of cardiac function by shifting cardiac apoptosis to autophagy in diabetic rabbits [13].Another study showed that RDN could decrease cardiomyocyte apoptosis in canines with prolonged atrial pacing [14]. However, how RDN and by what mechanisms regulates apoptosis in MI-HF is still unclear. Our study aimed to clarify the effect of RDN on cardiac function, and fibrosis, apoptosis-related factors and ER stress associated factors in HF dogs.

The present study in our lab demonstrated that RDN improved the deterioration of LV function.

and LV dilatation post-MI, including a significant increase in LVEF and a significant decrease in LVEDd compared with those in the model group. We further demonstrated that RDN decreased the level of NT-BNP, improved myocardial fibrosis, and decreased the expression of MMP-2 and MMP-9. Meanwhile, decreased Bax and caspase-3 mRNA and protein levels, and increased Bcl-2 mRNA and protein levels, led to an increase in the ratio of Bcl-2/Bax. Moreover, RDN significantly decreased the expression of GRP78 in HF dogs.

Myocyte apoptosis has been shown to commonly occur in HF, and the severity of myocardial apoptosis is closely associated with cardiac function. Numerous studies showed that ER stress (ERS) is an important pathway in cardiac myocyte apoptosis during the progression of HF [15]. GRP78, as a chaperone in the ER, plays a critical role in the regulation of ER dynamic balance, and is a key marker for the ER response. Our results show that RDN decreased GRP78 levels, indicating that RDN could reduce ER stress. Caspase, and the BCL-2 family of proteins are key factors in the apoptotic pathway [16]. Caspase 3 is one of the major caspases involved in apoptosis, which is regulated by the Bcl-2 family proteins. Bcl-2 can inhibit cell death, whereas Bax promotes apoptosis. Sympathetic nerve overactivity plays a critical role in ERS and cardiomyocyte apoptosis after MI. Norepinephrine (NE), an important neurotransmitter of the sympathetic nerve system, specifically stimulates the ß1-adrenergic receptor (AR), which induces ERS and apoptosis in cardiac myocytes in vitro and in vivo [17, 18]. Wang HJ et al. found that cardiac sympathetic afferent denervation attenuates cardiac remodeling and apoptosis and improves cardiovascular dysfunction in rats with HF [19]. Results of our TUNEL staining and apoptosis-associated protein and gene expression analyses also suggested that RDN treatment markedly reduced MI-induced cardiomyocyte apoptosis. Thus, we speculate that RDN improves cardiac function by reducing ERS and ERS mediated cardiomyocyte apoptosis.

Both myocyte apoptosis and non-myocyte apoptosis are involved in MI-induced HF [20]. Extracellular signals also modulate apoptosis in the myocardial matrix. Matrix metalloproteinases (MMPs), which are key substances in the cardiac interstitial matrix, increase after MI, and MMP levels correlate with the extent of cardiac dysfunction in HF patients [21]. MMPs regulate the remodeling process by facilitating extracellular matrix turnover and, therefore, play a fundamental role in tissue remodeling, including remodeling of the heart [22]. Two of the most widely studied cardiac MMPs are MMP-2 and MMP-9. The involvement of MMP-2 and MMP-9 is considered to be important, as MMP-2 and MMP-9 are capable of degrading interstitial fibrillar collagen and causing non-myocyte apoptosis, ultimately leading to systolic and diastolic impairment in the heart. In our study, we found that RDN significantly decreased cardiac MMP-2 and MMP-9 expression. Given the close relationship between MMPs and non-myocyte apoptosis, MMP-2 and MMP-9 may play a role in decreasing cardiac non-myocyte apoptosis.

## Conclusion

RDN improves cardiac function, possibly by reducing ERS, inhibiting cardiomyocyte apoptosis and decreasing MMPs levels.

### Limitations

The small sample size is a limitation of our study, We will further expand the experimental sample size to confirm our conclusion and further explore the effect of RDN on the ERS pathway. In addition, multicenter, randomized, controlled, double-blinded studies may be required to validate the efficacy and safety of RDN in patients with HF.

## Abbreviations

AI: Apoptotic index
AMI: Acute myocardial infarction
ELISA: Enzyme-linked immunosorbent assay
ERS: Endoplasmic reticulum stress
HF: Heart failure
HR: Heart rate
IHS: Immunohistochemical score
LVEDD: Left ventricular end-diastolic dimension
LVEDP: Left ventricular end-diastolic pressure
LVEF: Left ventricular ejection fraction
LVESD: Left ventricular end-systolic dimension
LVSP: Left ventricular systolic pressure
MMPs: Matrix metalloproteinases
NT-BNP: N-terminal pronatriuretic peptide
RDN: Renal sympathetic denervation
RT-PCR: Real-time reverse transcription polymerase chain reaction
